# Determinants of Integrated Management of Childhood Illness (IMCI) non–severe pneumonia classification and care in Malawi health facilities: Analysis of a national facility census

**DOI:** 10.7189/jogh.07.020408

**Published:** 2017-12

**Authors:** Emily White Johansson, Humphreys Nsona, Liliana Carvajal–Aguirre, Agbessi Amouzou, Helena Hildenwall

**Affiliations:** 1 Department of Women’s and Children’s Health, Uppsala University, Uppsala, Sweden; 2 Integrated Management of Childhood Illness (IMCI) Unit, Ministry of Health, Lilongwe, Malawi; 3 Data and Analytics Section, United Nations Children’s Fund, New York, New York, USA; 4 Global Health – Health Systems and Policy Research Group, Department of Public Health Sciences, Karolinska Institutet, Stockholm, Sweden

## Abstract

**Background:**

Research shows inadequate Integrated Management of Childhood Illness (IMCI)–pneumonia care in various low–income settings but evidence is largely from small–scale studies with limited evidence of patient–, provider– and facility–levels determinants of IMCI non–severe pneumonia classification and its management.

**Methods:**

The Malawi Service Provision Assessment 2013–2014 included 3149 outpatients aged 2–59 months with completed observations, interviews and re–examinations. Mixed–effects logistic regression models quantified the influence of patient–, provider and facility–level determinants on having IMCI non–severe pneumonia and its management in observed consultations.

**Findings:**

Among 3149 eligible outpatients, 590 (18.7%) had IMCI non–severe pneumonia classification in re–examination. 228 (38.7%) classified cases received first–line antibiotics and 159 (26.9%) received no antibiotics. 18.6% with cough or difficult breathing had 60–second respiratory rates counted during consultations, and conducting this assessment was significantly associated with IMCI training ever received (odds ratio (OR) = 2.37, 95% confidence interval (CI): 1.29–4.31) and negative rapid diagnostic test results (OR = 3.21, 95% CI: 1.45–7.13). Older children had lower odds of assessments than infants (OR = 48–59 months: 0.35, 95% CI: 0.16–0.75). Children presenting with any of the following complaints also had reduced odds of assessment: fever, diarrhea, skin problem or any danger sign. First–line antibiotic treatment for classified cases was significantly associated with high temperatures (OR = 3.26, 95% CI: 1.24–8.55) while older children had reduced odds of first–line treatment compared to infants (OR = 48–59 months: 0.29, 95% CI: 0.10–0.83). RDT–confirmed malaria was a significant predictor of no antibiotic receipt for IMCI non–severe pneumonia (OR = 10.65, 95% CI: 2.39–47.36).

**Conclusions:**

IMCI non–severe pneumonia care was sub–optimal in Malawi health facilities in 2013–2014 with inadequate assessments and prescribing practices that must be addressed to reduce this leading cause of mortality. Child’s symptoms and age, malaria diagnosis and provider training were primary influences on assessment and treatment practices. Current evidence could be used to better target IMCI training and support to improve pneumonia care for sick children in Malawi facilities.

Despite the enormous progress in child survival over the past two decades, approximately six million children under five years still die each year globally [1]. These deaths largely occur during the neonatal period or are due to infectious causes, such as pneumonia [2]. Indeed, pneumonia remains a leading cause of child mortality accounting for nearly one million under–five deaths annually. Early and effective treatment of childhood pneumonia is therefore a cornerstone of child survival programs [3].

Since the 1990s, WHO and UNICEF have promoted the Integrated Management of Childhood Illness (IMCI) strategy in low– and middle–income countries to effectively manage pneumonia and other common causes of child morbidity and mortality in an integrated manner [4]. While IMCI has great potential to improve health worker performance and quality care for sick children [5], poor implementation in routine practice has been documented in various settings over the past few decades [6,7]. This includes inadequate care for the IMCI–pneumonia algorithm in particular, which has been demonstrated in Malawi [8,9] although a more recent study from rural Malawi suggested stronger IMCI–pneumonia performance among service providers [10].

Yet this evidence is largely derived from small–scale studies in limited facility contexts without examination of determinants of IMCI–pneumonia classification or its case management. A national facility census, or Service Provision Assessment (SPA), was conducted in Malawi in 2013–2014 that included observed sick child consultations and re–examinations [11]. While an analysis of overall correct IMCI–pneumonia care is not possible using this facility census given limited observation and re–examination protocols (see Methods), it does provide a unique opportunity to identify determinants of having an IMCI non–severe pneumonia episode, such as child’s age or symptoms [12,13]. It also allows for a wide ranging assessment of patient–, provider– and facility–level predictors for conducting select assessments (60–second respiratory rate count) and prescribing first–line treatment to classified cases. Such evidence could help target IMCI training and support going forward in order to improve quality pneumonia care across Malawi health facilities.

## METHODS

### Study setting

Malawi is a low–income country in sub–Saharan Africa with an estimated population of 17 million [14]. Malawi’s under–five mortality rate declined from 242 deaths per 1000 live births in 1990 to 64 in 2015 [1], which achieved the Millennium Development Goal for child mortality. This significant reduction has been attributed to scaling–up interventions effective against the leading causes of child death, reducing child undernutrition and mother–to–child HIV transmission as well as improving quality childbirth care [15]. Prior to the Malawi Service Provision Assessment (SPA) in 2013–2014, IMCI guidelines were last updated in 2013 to reflect test–based malaria case management and wide–scale IMCI in–service training was previously implemented in 2009. Nationwide deployment of malaria rapid diagnostic tests (RDT) was initiated in July 2011 accompanied by training in RDT safety and use along with basic information on managing RDT–negative cases [16].

The Malawi health system generally includes both government facilities and publicly–supported facilities managed by the Christian Health Association of Malawi (CHAM) [16]. The three facility levels include health centers, district hospitals and regional hospitals. Health centers are the lowest level and deliver primary health care services that are generally led by a medical assistant or nurse midwife technician. District hospitals are referral facilities at the next level that provide in–patient care, laboratory diagnostics and maternity care that are generally led by medical doctors and clinical officers. Regional or central hospitals are the highest level that are generally research and teaching institutions that provide specialized medical care. Community treatment services are also available for sick children but were not included in this facility–based assessment.

### Survey methods

The Malawi Service Provision Assessment (SPA) was conducted in June 2013–February 2014 by the Ministry of Health and The DHS Program, which includes facility and laboratory audits, observed consultations with limited re–examination, patient exit interviews and health worker interviews. Survey methods are described elsewhere including procedures for obtaining ethical approval and participant consent [11].

**Figure 1:**
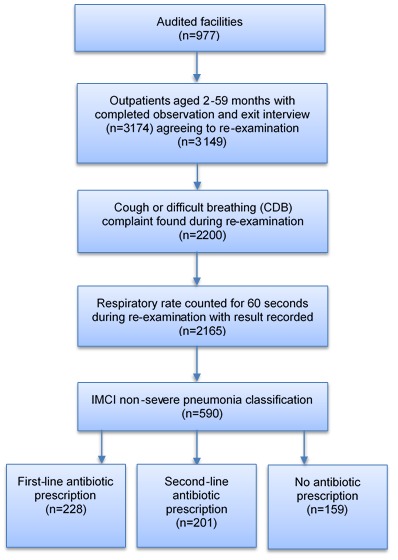
Study sample. Frequencies were weighted to account for the unequal probabilities of selection due to differing client volumes on the interview date. IMCI non–severe pneumonia classification is defined as cough or difficult breathing complaint and a respiratory rate of 50 breaths or more per minute (2 up to 12 months) or 40 breaths or more per minute (12 months up to 5 years) recorded during re–examination. First–line antibiotic prescription refers to benzyl penicillin injection or amoxicillin (capsule or syrup). Second–line antibiotic prescription refers to cotrimoxazole (syrup or tablet) or other antibiotic (injection, syrup or capsule). Antibiotic treatment totals sum to 588 observations (not 590) due to missing values.

Briefly, Malawi SPA 2013–2014 was designed as a census of all formal public and private facilities in the country to include 977 facilities out of 1060 on the Ministry of Health master facility list. Non–response was due to refusal (3%), closure (2%), inaccessibility (2%) or other issue (1%). At each facility, outpatients were systematically selected for observation based on the expected patient load for sick child curative services on the interview date in order to yield no more than 15 observations per facility. Outpatients were eligible to participate if they were less than five years old and presented with an illness complaint that was not an exclusive injury or non–disease condition. Children aged 2–59 months attending an observed outpatient consultation were included in this study if consent for the observed consultation, exit interview and re–examination were obtained. A total of 3149 observations met these criteria and were included in the analysis (**Figure 1**). A median of 3 observations were conducted at each facility.

During each observed sick child consultation, an observer was present to silently record whether certain IMCI assessments or examinations were completed, such as felt the child for fever or body hotness, counted breathing for 60 seconds or checked skin turgor for dehydration. However, there is no recording of assessment quality nor were all IMCI assessments included in the observer checklist, notably it was not recorded if the provider checked for chest in–drawing or asked about illness duration. After each consultation, the observer asked the provider to report all diagnoses/classifications and treatments prescribed to the sick child. A limited re–examination protocol was conducted during the exit interview and included measuring temperature, checking anemia symptoms and counting respiratory rates for 60 seconds if cough or difficult breathing (CDB) was reported. Both the observed consultation and re–examination was conducted by clinicians, nurses or nurse midwives trained in these specific protocols. For these reasons, it was not possible to directly assess correct pneumonia case management by comparing observed practices with ‘gold standard’ re–examination since neither the observation or re–examination provided complete information for such analyses. Nevertheless, the large number of facilities audited and broad data collection scope provide an opportunity to examine a wide–range of determinants for having a IMCI non–severe pneumonia episode, performing certain assessments and prescribing antibiotics for classified cases.

### Classification of IMCI non–severe pneumonia

IMCI non–severe pneumonia was defined in this study according to 2013 Malawi IMCI guidelines [17]. It was based on re–examination of the child as having a cough and difficult breathing (CDB) complaint and rapid breathing of 50 or more breaths per minute (2 up to 12 months) or 40 or more breaths per minute (12 to 59 months). IMCI severe pneumonia is classified based on chest in–drawing with or without fast breathing. However, chest in–drawing assessment was not part of re–examination or recorded as done in the observed consultation. For this reason, our analysis focuses on IMCI non–severe pneumonia since a raised respiratory rate *at least* indicates non–severe classification. In 2014, IMCI guidelines were updated to define non–severe pneumonia as fast breathing and/or chest in–drawing but this revision occurred after survey implementation and is not used in this paper [18].

### Assessment of IMCI non–severe pneumonia

IMCI non–severe pneumonia assessment was defined as a 60–second respiratory rate count observed and recorded during the consultation. This analysis was based on the subset of outpatients aged 2–59 months with CDB complaint reported in the exit interview.

### Treatment of IMCI non–severe pneumonia

IMCI non–severe pneumonia treatment was derived from provider reports of treatments prescribed to the sick child after the observation. First–line antibiotic treatment included benzyl penicillin injection or amoxicillin (capsule or syrup) since it was not possible to differentiate severe and non–severe cases in this data set. Second–line antibiotic treatment included cotrimoxazole (syrup or tablet) or other antibiotic (injection, syrup or capsule). Hierarchical coding assigned the most appropriate prescription to the observation if multiple drugs were prescribed. This analysis was based on the subset of outpatients aged 2–59 months with IMCI non–severe pneumonia that attended a facility with amoxicillin available on the interview date. Only 13 IMCI non–severe pneumonia cases (unweighted) were observed in facilities without amoxicillin and these observations were removed from this analysis.

### Explanatory variables

Patient–, provider and facility–level variables with potential to affect IMCI–pneumonia classification and care were explored in these analyses [8–10,19–25]. Patient–level variables included child’s sex and age (2–11, 12–23, 24–35, 36–47, 48–59 months), caregiver’s age (Under 20, 20–29, 30–39, 40 or more years), caregiver’s education (none, primary or at least secondary attendance), RDT conducted prior to or during the initial consultation according to provider reports, and if so, reported RDT results (positive or negative), child’s temperature (37.5 or less, 37.6–38.9 or 39.0–40.8) recorded in re–examination, illness duration (0–1, 2–4, 5 or more days), consultation start hour (7–10 am, 11–2 pm, 3–5 pm) recorded in the observation, reported wait time (under 10, 11–30, 31–60, 60 or more minutes), first or follow–up visit for the current illness, and symptom complaints (fever, diarrhea, ear problem, eye problem, skin problem or any danger sign). Any danger sign includes vomits everything, convulsions, lethargy or inability to eat, drink or breastfeed. Patient–level variables were reported by the caregiver during the exit interview unless otherwise noted.

Provider–level variables included job qualification (doctor, medical assistant, nurse or other provider) and year qualification received (before 2000, 2001–2009, 2010 to present), provider sex, supervisor or in–charge status, IMCI in–service training ever received or not and recent supervisory visit (none, within past 3 months, over 3 months ago). Provider–level variables were reported during the provider interview.

Facility–level variables included facility type (hospital or other), managing authority (government or CHAM/other private), total staff doctors (0, 1, 2+ doctors), routine general user fees or not, routine management meetings reportedly occur or not, region (north, central, south), location (urban, rural), timer available, amoxicillin or any antibiotic available, as well as malaria risk (infection prevalence) values for 2013–2014 linked to data sets through geocoded facility locations and transmission season estimates derived from facility locations and interview date [26,27]. Facility–level variables were recorded during the facility audit unless otherwise noted.

### Missing values

Observations with missing values were removed from analyses using listwise deletion. For the IMCI non–severe pneumonia classification analysis, 158 (5%) observations were dropped that had missing values for at least one explanatory variable. For the assessment and treatment analyses, 65 (3%) and 9 (2%) observations were removed respectively.

### Data analyses

Mixed–effects logistic regression models quantified the influence of explanatory variables on the binary outcomes of interest. Variables were included as categorical fixed effects nested within facility identifiers and normal distribution of the random effects was assumed. Bivariate analyses were initially conducted for each variable to estimate crude odds ratios separately for each of the outcomes. Variables found to be statistically significant at the 0.1 level in bivariate analyses were subsequently included in final models to obtain adjusted odds ratios. Variance inflation factors were used to detect multi–collinearity among variables prior to inclusion in final models. Given the importance of child’s age in model outputs, we tested for an interaction between child’s age and IMCI training on the assessment and treatment outcomes in final models. Results were stratified to examine effect differences across age groups (2–11, 12–23, 24–35, 36–47, 48–59 months). Point estimates were calculated using weights to account for unequal probabilities of selection due to differing client volumes at facilities on the interview date. Standard error estimation accounted for clustering of client observations within facilities. The level of statistical significance was set to 0.05. Stata 13.1 (Stata Corp., College Station, TX) was used for analyses.

## RESULTS

Among 3149 eligible outpatients aged 2–59 months, 590 (18.7%) were classified with IMCI non–severe pneumonia in re–examination (**Table 1**). Among these classified cases, 228 (38.7%) received benzyl penicillin injection or amoxicillin, 157 (26.6%) received cotrimoxazole, 44 (7.5%) received other antibiotics and 159 (26.9%) received no antibiotic (**Figure 1**). Among 2271 outpatients aged 2–59 months with CDB complaints reported in the exit interview, 422 (18.6%) had a 60–second respiratory rate counted in the consultation (**Online Supplementary Document(Online Supplementary Document)**).

**Table 1:** Characteristics of outpatients aged 2–59 months with IMCI non–severe pneumonia, Malawi health facilities, 2013–2014*

		Outpatients aged 2–59 months (No.)	IMCI non–severe pneumonia (No.)	Percent IMCI non–severe pneumonia (95% CI)
**Total**		**3149**	**590**	**18.7 (16.6–21.1)**
Fever complaint	Yes	2110	397	18.8 (16.4–21.5)
	No	962	164	17.0 (13.5–21.3)
Diarrhea complaint	Yes	899	166	18.5 (15.2–22.3)
	No	2249	423	18.7 (16.3–21.5)
Danger sign complaint	Yes	1481	283	19.1 (16.3–22.4)
	No	1669	307	18.4 (15.7–21.4)
RDT result	Positive	378	78	20.6 (15.1–27.5)
	Negative	653	151	23.2 (18.8–28.1)
Temperature (Celsius)	37.5 or less	2436	410	16.8 (14.5–19.3)
	37.6–38.9	595	144	24.3 (19.9–29.2)
	39.0–40.8	99	30	30.6 (21.7–41.3)
Child’s age (months)	2–11	1124	166	14.7 (11.5–18.7)
	12–23	912	261	28.6 (24.6–33.0)
	24–35	540	88	16.2 (12.6–20.7)
	36–47	317	39	12.2 (8.4–17.3)
	48–59	257	37	14.5 (9.6–21.2)
Illness duration (days)	0–1	726	135	18.6 (15.0–22.7)
	2–4	1992	363	18.2 (15.7–21.0)
	5 or more	420	92	22.0 (17.0–28.0)
Malaria endemicity (PfPR_2–10_)	Under 0.20	2367	448	18.9 (16.4–21.6)
	0.20–0.39	782	142	18.2 (14.4–22.7)
Transmission season	Peak	428	68	15.9 (12.3–20.3)
	Off–peak	2721	522	19.2 (16.8–21.8)
Residence	Urban	1007	170	16.8 (12.4–22.5)
	Rural	2142	420	19.6 (17.5–22.0)
Region	North	463	68	14.7 (11.5–18.7)
	Central	1583	317	20.0 (17.2–23.1)
	South	1103	205	18.4 (14.6–23.4)
Any antibiotic observed	Yes	3142	589	18.6 (16.5–20.9)
	No	3	<1	25.0 (––)
Amoxicillin observed	Yes	3094	580	18.7 (16.6–21.1)
	No	52	10	18.8 (9.9–33.0)
Facility type	Hospital (central, district, rural, other)	1136	200	17.6 (13.1–23.2)
	Other facility type	2014	390	19.4 (17.4–21.5)
Managing authority	Government	2404	454	18.9 (16.3–21.8)
	CHAM or other private ownership	745	136	18.2 (15.2–21.8)

IMCI – Integrated Management of Childhood Illness, CI – confidence interval, RDT – rapid diagnostic test, PfPR – *Plasmodium falciparum* parasite rate, CHAM – Christian Health Association of Malawi

*Outpatients aged 2–59 months with completed observations, exit interviews and re–examinations were included. IMCI non–severe pneumonia classification was identified in re–examination based on CDB complaint and a 60–second respiratory rate count of 50 or more breaths per minute (2 up to 12 months) and 40 or more breaths per minute (12 months to 5 years). Frequencies and cross–tabulations were weighted to account for the unequal probabilities of selection due to differing client volumes on the interview date.

### IMCI non–severe pneumonia classification

**Table 2** indicates a significant association between child’s age, raised temperature and illness duration on having IMCI non–severe pneumonia classification. Compared to infants, there was nearly three times higher odds of having this classification among 12–23 months (odds ratio (OR) = 2.87, 95% confidence interval (CI): 2.17–3.78) while there was no statistical difference with older age groups. Outpatients with a moderate or high temperature had 1.59 (95% CI: 1.21–2.09) and 2.38 (95% CI: 1.41–4.04) times higher odds of receiving this classification respectively than those with no or low temperature. Compared to a short illness duration (0–1 day), outpatients reporting illness lasting 5+ days had 1.57 (95% CI: 1.08–2.27) times higher odds of having IMCI non–severe pneumonia.

**Table 2:** Determinants of IMCI non–severe pneumonia classification in outpatients aged 2–59 months, Malawi health facilities, 2013–2014*

			Adjusted OR	95% CI	*P*
**Patient**	Fever complaint	No	1.00		
		Yes	1.07	0.83–1.38	0.616
	Diarrhea complaint	No	1.00		
		Yes	0.80	0.62–1.03	0.090
	Danger sign complaint	No	1.00		
		Yes	1.09	0.87–1.37	0.438
	Temperature (Celsius)	37.5 or less	1.00		
		37.6–38.9	1.59	1.21–2.09	0.001
		39.0–40.8	2.38	1.41–4.04	0.001
	Child’s age (months)	2–11	1.00		
		12–23	2.87	2.17–3.78	<0.001
		24–35	1.25	0.89–1.76	0.192
		36–47	0.94	0.62–1.45	0.794
		48–59	1.05	0.67–1.66	0.824
	Illness duration (days)	0–1	1.00		
		2–4	1.08	0.82–1.43	0.571
		5 or more	1.57	1.08–2.27	0.016
**Facility**	Malaria endemicity (PfPR_2–10_)	Less than 0.20	1.00		
		0.20–0.39	0.76	0.52–1.10	0.148
	Transmission season	Peak	1.00		
		Off–peak	0.89	0.61–1.29	0.538
	Residence	Urban	1.00		
		Rural	1.26	0.87–1.82	0.225
	Region	North	1.00		
		Central	1.48	0.98–2.24	0.061
		South	0.91	0.61–1.35	0.623
	Facility type	Hospital (central, district, rural, other)	1.00		
		Other facility type	1.06	0.71–1.59	0.769
	Managing authority	Government	1.00		
		CHAM or other private ownership	0.93	0.69–1.26	0.654

IMCI – Integrated Management of Childhood Illness, CI – confidence interval, OR – odds ratio, PfPR – Plasmodium falciparum parasite rate, CHAM – Christian Health Association of Malawi

*Variables presented in this table were significant (*P* < 0.1) in bivariate analyses and were then included simultaneously in the final model to obtain adjusted odds ratios. Mixed–effects logistic regression models quantified the influence of the above variables on receiving IMCI non–severe pneumonia classification (or not) adjusted for data clustering.

### Count respiratory rates for 60 seconds

**Table 3** shows the significance of child’s age, symptoms, IMCI in–service training, provider qualification, region and reported wait time on the odds of receiving a 60–second respiratory rate count to assess IMCI non–severe pneumonia. There was a significant and consistent decline in assessment odds with increasing age of the child. Compared to infants, the odds of receiving a 60–second respiratory rate count declined by 38% for 12–23 months (OR = 0.62, 95% CI: 0.49–0.95), by 62% for 24–35 months (OR = 0.38, 95% CI: 0.22–0.65), by 47% for 36–47 months (OR = 0.53, 95% CI: 0.27–1.05) and by 65% for 48–59 months (OR = 0.35, 95% CI: 0.16–0.75).

**Table 3:** Determinants of taking a 60–second respiratory rate count in outpatients aged 2–59 months with cough or difficult breathing complaints, Malawi health facilities, 2013–2014*

			Adjusted OR	95% CI	*P*
**Patient**	Fever complaint	No	1.00		
		Yes	0.62	0.43–0.95	0.018
	Diarrhea complaint	No	1.00		
		Yes	0.61	0.43–1.01	0.023
	Skin problem complaint	No	1.00		
		Yes	0.21	0.07–0.64	0.006
	Any danger sign complaint	No	1.00		
		Yes	0.70	0.49–1.02	0.052
	Child’s age (months)	2–11	1.00		
		12–23	0.62	0.49–0.95	0.030
		24–35	0.38	0.22–0.65	<0.001
		36–47	0.53	0.27–1.05	0.070
		48–59	0.35	0.16–0.75	0.007
	Caregiver’s age (years)	11–19	1.00		
		20–29	0.64	0.35–1.14	0.125
		30–39	1.20	0.61–2.27	0.627
		40 or older	0.33	0.10–1.14	0.080
	Wait time (minutes)	10 or less	1.00		
		11–30	0.42	0.22–0.81	0.009
		31–59	0.74	0.41–1.32	0.303
		60 or more	1.00	0.56–1.75	0.955
	RDT results†	Positive	1.00		
		Negative	3.21	1.45–7.13	0.001
**Facility**	Malaria endemicity (PfPR_2–10_)	Less than 0.20	1.00		
		0.20–0.39	1.25	0.60–2.61	0.630
	Region	North	1.00		
		Central	1.15	0.50–2.60	0.749
		South	0.31	0.13–0.71	0.006
	Doctors (total on staff)	0	1.00		
		1	1.40	0.38–5.49	0.593
		2 or more	0.33	0.09–1.17	0.085
	IMCI guidelines available	No	1.00		
		Yes	1.19	0.64–2.32	0.546
**Provider**	Qualification	Doctor	1.00		
		Medical assistant	0.78	0.37–1.80	0.607
		Nurse or other provider	0.33	0.12–1.01	0.051
	Supervisor or in–charge	No	1.00		
		Yes	1.29	0.68–2.46	0.425
	Qualification received (year)	Before 2000	1.00		
		2000–2009	1.15	0.56–2.33	0.713
		2010 to present	0.69	0.30–1.60	0.394
	IMCI in–service training (ever received)	No	1.00		
		Yes	2.37	1.29–4.31	0.006

CI – confidence interval, OR – odds ratio, RDT – rapid diagnostic test, PfPR – Plasmodium falciparum parasite rate, CHAM – Christian Health Association of Malawi, IMCI – Integrated Management of Childhood Illness

*Table S1 in **Online Supplementary Document(Online Supplementary Document)** presents descriptive statistics for outpatients aged 2–59 months with CDB complaints reported in exit interviews (n = 2271). CI refers to confidence interval. Variables presented in this table were significant (*P* < 0.1) in bivariate analyses and were then included simultaneously in the final model to obtain adjusted odds ratios. Mixed–effects logistic regression models quantified the influence of the above variables on counting respiratory rates for 60 s (or not) adjusted for data clustering.

†RDT results is based on a subset analysis of outpatients with CDB complaints and reported RDT results (n = 692).

Reporting other symptoms with CDB complaint significantly reduced the odds of having a 60–second respiratory rate counted in the consultation. Assessment odds declined by 38% if fever was reported (OR = 0.62, 95% CI: 0.43–0.95) compared to not reported, by 39% if diarrhea was reported (OR = 0.61, 95% CI: 0.43–1.01), by 79% for reported skin problems (OR = 0.21, 95% CI: 0.07–0.64), and by 30% if any danger sign was reported although the latter was not a statistically significant reduction (OR = 0.70, 95% CI: 0.49–1.02).

Outpatients attended by providers that ever received IMCI in–service training had 2.37 times higher odds of receiving a 60–second respiratory rate count (95% CI: 1.29–4.31) than those seen by providers without such training. Compared to doctors, clients attended by nurses, midwives or other lower–level providers had reduced assessment odds (OR = 0.33, 95% CI: 0.12–1.01) although there was no significant difference with medical assistants. Attendance at facilities in the South region was associated with significantly lower assessment odds (OR = 0.31, 95% CI: 0.13–0.71) than in the North region. In a subset analysis of outpatients with CDB complaints and reported RDT results (n = 692), RDT–negative cases had 3.21 times higher assessment odds than outpatients with RDT–confirmed malaria (95% CI: 1.45–7.13) in the adjusted analysis.

There was also evidence of an interaction between categorical variables child’s age and IMCI training (p–values ranged from 0.006 to 0.813) on the assessment outcome. To further explore this result, the final model was stratified by age groupings. Outpatients aged 12–23 months (OR = 9.56, 95% CI: 3.03–30.18) and 48–59 months (OR = 261.97, 95% CI: 1.46–47281.50) that visited providers ever receiving IMCI in–service training had significantly higher assessment odds than outpatients seen by providers with no such training although effect sizes should be interpreted with caution due to few observations and positive outcomes (**Table 4**). There was a negligible difference between those visiting trained and untrained providers in other age groups. There was also limited overlap in confidence intervals of the adjusted odds ratios for 2–11 and 12 to 23–month–olds suggesting a difference across these age groups in the effect of training on assessment odds. Stratified models for the treatment outcome showed no significant difference in IMCI training across age groupings (data not shown).

**Table 4:** Effect of IMCI training on counting respiratory rates for 60 s across age groupings, Malawi health facilities, 2013–2014*

	IMCI in–service training (ever received or not)	Adjusted OR	95% CI	*P*
2–11 months	No	1.00		
	Yes	1.50	0.70–3.24	0.301
12–23 months	No	1.00		
	Yes	9.56	3.03–30.18	<0.001
24–35 months	No	1.00		
	Yes	2.06	0.60–7.07	0.250
36–47 months	No	1.00		
	Yes	2.04	0.35–11.85	0.429
48–59 months	No	1.00		
	Yes	261.97	1.46–47 281.50	0.036

IMCI – Integrated Management of Childhood Illness, CI – confidence interval, OR – odds ratio

*Mixed–effects logistic regression models quantified the influence of IMCI in–service training on counting respiratory rates for 60 s (or not) across each age group adjusted for variables listed in **Table 3** and data clustering. Results for the children aged 48–59 months should be interpreted with caution due to few observations and positive outcomes.

### Antibiotic prescriptions

**Figure 2:**
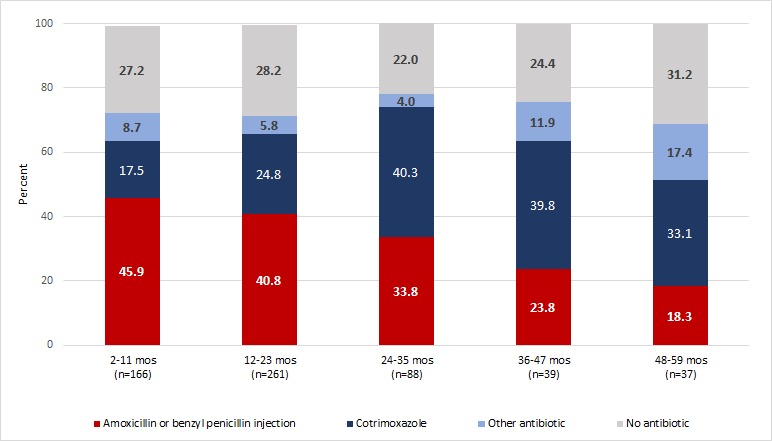
Antibiotic prescriptions for IMCI non–severe pneumonia by age groups, Malawi health facilities, 2013–2014. Frequencies were weighted to account for the unequal probabilities of selection due to differing client volumes on the interview date.

**Figure 2** depicts antibiotic prescriptions for IMCI non–severe pneumonia cases across different age groups. Nearly half (45.9%) of infants with IMCI non–severe pneumonia received first–line antibiotics and this proportion consistently declined with older ages to a low of 18.3% among 48 to 59–month–olds. In contrast, second–line antibiotics (cotrimoxazole or other antibiotic) were more often prescribed to older children with 50.5% of 48 to 59–month–olds incorrectly treated compared to 26.2% of infants. The proportion of outpatients receiving no antibiotic for IMCI non–severe pneumonia ranged from 22.0% among 24 to 35–month–olds to 31.2% among those 48–59 months old.

First–line antibiotic treatment of classified cases was significantly associated with younger ages, high temperatures and more staff doctors (**Table 5**). Compared to infants, odds of first–line treatment declined by 67% for ages 36–47 months (OR = 0.33, 95% CI: 0.12–0.88) and by 71% for ages 48–59 months (OR = 0.29, 95% CI: 0.10–0.83) while there was no statistical difference with other age groups. Outpatients with high temperatures (39.0°C or more) had 3.26 times higher odds of receiving first–line antibiotics than those with low or no temperature (95% CI: 1.24–8.55). Children with IMCI non–severe pneumonia attending facilities with two or more staff doctors had 2.90 times higher odds of receiving first–line antibiotic treatment than those visiting facilities with no staff doctors (95% CI: 1.22–6.88).

**Table 5:** Determinants of first–line antibiotic treatment for IMCI non–severe pneumonia in outpatients aged 2–59 month, Malawi health facilities, 2013–2014*

			Adjusted OR	95% CI	*P*
**Patient**	Temperature (Celsius)	37.5 or less	1.00		
		37.6–38.9	1.17	0.69–1.97	0.569
		39.0–40.8	3.26	1.24–8.55	0.016
	Child’s age (months)	2–11	1.00		
		12–23	0.83	0.48–1.43	0.502
		24–35	0.62	0.31–1.25	0.183
		36–47	0.33	0.12–0.88	0.027
		48–59	0.29	0.10–0.83	0.021
**Facility**	Transmission season	Peak	1.00		
		Off–peak	0.61	0.29–1.27	0.187
	Region	North	1.00		
		Central	1.87	0.88–3.97	0.103
		South	0.94	0.45–1.98	0.875
	Total staff doctors	0	1.00		
		1	0.58	0.18–1.89	0.264
		2 or more	2.90	1.22–6.88	0.016

IMCI – Integrated Management of Childhood Illness, OR – odds ratio, CI – confidence interval

*Table S2 in **Online Supplementary Document(Online Supplementary Document)** presents descriptive statistics for outpatients aged 2–59 mo with IMCI non–severe pneumonia classification receiving first–line antibiotic treatment (n = 590). CI refers to confidence interval. Variables presented in this table were significant (*P* < 0.1) in bivariate analyses and were subsequently included simultaneously in the final model to obtain adjusted odds ratios. Mixed–effects logistic regression models quantified the influence of variables on first–line antibiotic treatment (or not) adjusted for data clustering. A total of 13 observations (unweighted) with IMCI non–severe pneumonia attended facilities without amoxicillin available on the interview date and these observations were removed from this analysis. Analyses for no antibiotic prescription for IMCI non–severe pneumonia found only diarrhea significantly associated with no treatment in the bivariate analyses (crude OR = 1.80, 95% CI: 1.08–3.01). In the subset analysis among sick child clients aged 2–59 mo with IMCI non–severe pneumonia and RDT results (n = 216), RDT–positive cases had 10.65 times higher odds of no antibiotic prescription than RDT–negative cases (adjusted OR = 10.65, 95% CI: 2.39–47.36).

In contrast, those with IMCI non–severe pneumonia who also presented with a diarrhea complaint had significantly higher odds of no antibiotic treatment in the bivariate analysis (OR = 1.80, 95% CI: 1.08–3.01) while no other significant bivariate associations were identified. Among classified cases with RDT results (n = 216), RDT–positive cases had significantly higher odds of no antibiotic receipt than RDT–negative cases (95% CI: 2.39–47.36) although the effect size should be interpreted with caution due to few observations and positive outcomes.

## DISCUSSION

Overall, there was sub–optimal IMCI–pneumonia care in Malawi health facilities in 2013–2014 in terms of completed assessments and antibiotic prescriptions for non–severe cases. Child’s symptoms and age, malaria diagnosis and provider training were main influences on assessment and treatment practices in this study, which could help inform IMCI training and support to improve pneumonia care for sick children.

In this study, IMCI non–severe pneumonia classification was significantly associated with raised temperature, child’s age and illness duration that are biologically plausible results and consistent with findings from other research [12,13,28]. While previous studies have shown strong correlations between measured or reported fever and IMCI–pneumonia classification, our findings indicate that nearly 1 in 5 (17%) children without a measured fever (37.5°C or less) had IMCI non–severe pneumonia classification in re–examination. This result is higher than expected and may potentially reflect rapid breathing over–diagnosis in re–examination since misclassification can occur even among trained providers [29].

Few patients with CDB complaints had a 60–second respiratory rate counted in the observation, which is consistent with research from Malawi and other settings [5,6,8]. Assessment for IMCI–pneumonia occurred less often if other symptoms were reported (fever, diarrhea, skin problems or any danger sign) or for RDT–confirmed malaria cases as well as among older children, those living in the South region or outpatients attending providers without IMCI in–service training or with lower job qualifications. Other research in both African and Asian settings has shown that malaria diagnosis or reporting other symptom complaints reduced correct IMCI–pneumonia management [23,30,31]. It is critical that IMCI training reinforce the importance of assessing outpatients for multiple conditions given symptom overlap and common co–morbidities particularly among the sickest children at highest risk of death [32].

Some evidence also suggests that IMCI training may improve health worker skills reinforcing current findings although the long–term impact of training programs on performance has been debated [33,34]. Our results also indicate poor assessment performance among lower–level providers that has been shown in other research from Malawi, and enhanced IMCI support may need to specifically target these providers [23]. Poorer IMCI–pneumonia assessment in the South region compared to the North has not been previously reported to our knowledge but is consistent with greater child mortality reductions found in the North than the South region in 2000–2013 [15].

There was a significant and consistent decline in counting respiratory rates with increasing age of the child that has been shown in other settings [35], and could reflect more clinical probing of infants given higher mortality rates in this age group. There may also be higher suspicion of bacterial infections in infants that could also drive higher assessment rates as suggested in qualitative research [24]. Yet while children aged 12–23 months are less often assessed for IMCI–pneumonia than infants, our findings indicate higher rates of IMCI non–severe pneumonia classification in this age group. This disconnect should be addressed through enhanced IMCI training and support, particularly since our stratified analysis suggests IMCI training may potentially have greater effect on raising assessment rates in this older age group. Beyond the clinical setting, the identified under–classification and under–treatment of pneumonia cases may also be informative for regional and global burden of disease estimates more broadly [2].

Common mistreatment of IMCI non–severe pneumonia cases was also found in this analysis. Few (39%) cases were prescribed first–line antibiotics while 27% classified cases received no antibiotic prescription despite attendance at facilities with available amoxicillin. Poor antibiotic targeting for IMCI non–severe pneumonia closely follows poor assessments for antibiotic need as previously discussed. First–line antibiotic prescription for classified cases was associated with raised temperature, child’s age and total staff doctors while receipt of no antibiotic prescription was associated with diarrhea complaint and RDT–confirmed malaria. Importantly, there was a significant and consistent decline in first–line treatment with increasing age of the child that also occurred with IMCI–pneumonia assessment. In addition, among older children with IMCI–pneumonia, second–line treatment (cotrimoxazole or other antibiotic) was more often prescribed even with amoxicillin available in the facility. This could suggest prioritization of first–line treatment for younger children at higher risk of dying or where there may be greater suspicion of bacterial infection. Higher temperatures also seemed to decrease the likelihood of pneumonia assessment while increasing the potential for antibiotic prescriptions suggesting sicker children are prioritized for treatment regardless of assessed need.

Results should be viewed in light of some data limitations. First, health workers may perform better during observations and there could be worse IMCI–pneumonia care in routine practice although this effect could wane over repeated observations [36,37]. Second, the observation protocol does not record all assessments in the IMCI pneumonia algorithm such as looking for wheeze, chest in–drawing or stridor that are needed to classify severe pneumonia. The quality of the assessment is also not recorded such as whether breathing rates were correctly counted in a calm child for 60 seconds while using a timer. Third, the re–examination protocol was limited to a 60–second respiratory rate count, measured temperature and recorded signs of anemia given time and staff constraints. Other signs of severe pneumonia were not assessed such as chest in–drawing or hypoxia nor were there assessments of general danger signs indicating severe disease requiring urgent attention. For these reasons, this study could not assess correct IMCI–pneumonia management overall including differentiation among severe from non–severe cases, nor the full IMCI protocol more broadly. Also, we did not assess the new IMCI pneumonia algorithm defining non–severe pneumonia as fast breathing and/or chest in–drawing. It is possible that chest in–drawing could improve pneumonia classification although some children with IMCI–pneumonia will not present with in–drawing making current results of continued importance. It is also possible that chest in–drawing assessment could be similarly associated with child’s age or other symptoms as found in this study. Fourth, rapid breathing can be difficult to assess even by trained providers leading to misclassification in either direction [29]. Finally, the IMCI algorithm was designed to include deliberate over–treatment of targeted conditions and recent studies have found IMCI–pneumonia is commonly classified in children who do not have x–ray confirmed disease [38,39]. Many outpatients with IMCI–pneumonia in this analysis likely do not need antibiotics although IMCI guidelines specify antibiotic treatment for presence of cough and fast breathing. The current lack of diagnostics to identify those in need of antibiotics will sustain continued misdirected treatment practices.

## CONCLUSIONS

Based on a national facility census including 3149 observed sick children aged 2–59 months, study findings indicate sub–optimal care for IMCI non–severe pneumonia in terms of completed assessments and antibiotic prescriptions. Few classified cases received first–line antibiotics and counting respiratory rates was not often conducted. Results reinforce the primary importance of child’s symptoms and age, malaria diagnosis as well as provider qualification and training on IMCI–pneumonia care in Malawi health facilities. Enhanced training and support is needed improve IMCI implementation particularly for the pneumonia algorithm, and current results suggest ways to better target these programs in the future. Greater focus on improved IMCI pneumonia care is urgently needed given its major contribution to child mortality in Malawi and globally.
