# Supplementary material for: Determinants of Integrated Management of Childhood Illness (IMCI) non–severe pneumonia classification and care in Malawi health facilities: Analysis of a national facility census
Source: J Glob Health. 2017 Nov 9;7(2):020408. doi: 10.7189/jogh.07.020408 (PMC5680530; doi:10.7189/jogh.07.020408)
Supplement: Online Supplementary Document [file jogh-07-020408-s001.pdf]

Online Supplementary Document

Johansson et al. Determinants of Integrated Management of Childhood Illness (IMCI) non–severe pneumonia classification and care in Malawi health facilities: Analysis of a national facility census

J Glob Health 2017;7:020408

Table S1: Characteristics of outpatients aged 2-59 months with cough or difficult breathing complaints with a 60-second respiratory rate count taken in the observed consultation, Malawi health facilities, 2013-2014

		N sick child clients 2-59 months with CDB complaint	N 60-second respiratory rate count	% 60-second respiratory rate count
	**Total**	**2271**	**422**	**18.6 (15.9-21.6)**
**Patient or consultation characteristics**				
Fever complaint	No	673	137	20.4 (16.1-25.6)
	Yes	1,541	269	17.5 (14.6-20.7)
Diarrhea complaint	No	1,653	334	20.2 (17.1-23.6)
	Yes	617	88	14.3 (11.3-18.0)
Ear problem complaint	No	2,244	417	18.6 (15.9-21.6)
	Yes	27	5	17.1 (6.7-37.1)
Eye problem complaint	No	2,215	413	18.7 (16.0-21.7)
	Yes	56	8	15.1 (7.7-27.5)
Skin problem complaint	No	2,188	414	18.9 (16.2-22.0)
	Yes	83	8	9.6 (4.4-19.7)
Any danger sign complaint	No	1,236	242	19.6 (16.4-23.2)
	Yes	1,035	180	17.4 (14.3-21.0)
RDT done prior to consultation	No	1,540	266	17.2 (14.3-20.7)
	Yes	731	156	21.4 (17.2-26.3)
RDT result	Positive	245	33	13.3 (8.4-20.5)
	Negative	464	115	24.8 (19.2-31.4)
Temperature (Celsius)	37.5 or less	1,763	319	18.1 (15.3-21.3)
	37.6 – 38.9	409	80	19.5 (14.8-25.2)
	39.0 – 40.8	69	11	16.7 (8.7-29.5)
Child sex	Male	1,145	204	17.8 (14.7-21.4)
	Female	1,126	218	19.4 (16.1-23.1)
Child age (months)	2 – 11	892	200	22.4 (18.5-26.8)
	12 – 23	657	106	16.2 (12.6-20.5)
	24 – 35	363	60	16.6 (12.0-22.5)
	36 – 47	204	33	16.2 (10.6-23.8)
	48 – 59	154	23	14.7 (9.3-22.4)
Caregiver age (years)	11 – 19	241	52	21.4 (15.3-29.2)
	20 – 29	1,297	219	16.9 (14.1-20.0)
	30 – 39	574	121	21.1 (16.5-26.7)
	40 or more	74	12	16.3 (9.0-27.6)
Caregiver education	None	260	40	15.6 (11.3-21.1)
	Primary	1,461	280	19.2 (16.2-22.5)
	Secondary or more	549	100	18.3 (13.3-24.6)
Consultation start hour	7am – 10am	1,743	325	18.7 (15.6-22.2)

	11am – 2 pm	419	91	21.8 (15.8-29.3)
	3pm – 5 pm	109	5	4.9 (1.9-12.2)
Wait time reported (minutes)	10 or less	494	89	18.0 (13.5-23.4)
	11 – 30	336	47	14.1 (10.1-19.3)
	31 – 60	436	78	17.9 (13.8-22.8)
	60 or more	858	177	20.7 (16.6-25.4)
	Don't know	148	31	20.8 (14.4-29.1)
Illness duration (days)	0 – 1	479	88	18.3 (13.8-23.9)
	2 – 4	1,494	274	18.3 (15.2-21.9)
	5 or more	293	59	20.1 (15.0-26.4)
First visit for current illness	First visit	2,113	399	18.9 (16.1-22.0)
	Follow-up visit	113	18	15.8 (10.0-24.0)
**Facility characteristics**				
Malaria endemicity ($PfPR_{2-10}$)	Under 0.20	1646	266	16.2 (13.3-19.4)
	0.20 – 0.39	625	156	25.0 (19.1-31.9)
Transmission season	Peak	2003	374	18.7 (15.8-22.0)
	Off-peak	267	48	17.8 (12.2-25.4)
Residence	Urban	730	88	12.0 (8.1-17.4)
	Rural	1541	334	21.7 (18.5-25.3)
Facility type	Hospital (central, district, rural, other)	842	127	15.0 (10.6-20.9)
	Other facility type	1428	295	20.7 (17.7-24.0)
Managing authority	Government	1752	324	18.5 (15.3-22.2)
	CHAM or other private	519	98	18.8 (14.4-24.2)
Region	North	330	68	20.6 (14.3-28.8)
	Central	1224	272	22.2 (18.0-27.2)
	South	717	82	11.4 (8.4-15.3)
Any antibiotic available	No	3	0	0.0
	Yes	2266	422	18.6 (15.9-21.6)
Amoxicillin available	No	39	1	2.8 (0.5-13.5)
	Yes	2230	421	18.9 (16.1-21.9)
Timer available	No	127	19	15.2 (6.8-30.5)
	Yes	2143	403	18.8 (16.0-21.9)
Total staff doctors	0	1539	343	22.3 (19.3-25.7)
	1	108	18	17.2 (8.8-30.8)
	2 – 4	346	39	1.2 (5.5-21.6)
	5 or more	278	21	7.7 (3.1-18.0)
User fees (routine, general)	No	1613	308	19.1 (15.8-22.9)
	Yes	658	114	17.3 (13.0-22.7)
IMCI guidelines available	No	642	100	15.6 (11.8-20.5)
	Yes	1629	322	19.7 (16.4-23.6)
**Provider characteristics**				
Job qualification	Doctor	261	41	15.7 (10.0-23.7)
	Medical assistant	1705	341	20.0 (16.7-23.8)
	Nurse or other lower-level provider	305	40	13.0 (8.5-19.3)
Year qualification received	Before 2000	415	80	19.4 (13.2-27.6)
	2000 – 2009	811	205	25.3 (20.9-30.3)
	2010 to present	978	132	13.5 (9.9-18.2)
Provider sex	Male	1447	295	20.4 (16.9-24.4)
	Female	757	123	16.2 (11.4-22.4)
Supervisor or in-charge	No	1064	144	13.5 (10.1-17.8)
	Yes	1139	274	24.1 (20.5-28.1)
IMCI in-service training	None	1117	145	13.0 (10.2-16.5)
	Ever received	1087	273	25.1 (20.6-30.2)
Recent supervision	None	397	69	17.4 (11.7-25.1)

Within past 3 months	1465	272	18.5 (15.1-22.5)
Over 3 months ago	342	77	22.6 (16.7-29.8)

Table S2: Characteristics of outpatients aged 2-59 months with IMCI non-severe pneumonia receiving antibiotic treatment by type, Malawi health facilities, 2013-2014

		N outpatients 2-59 months with IMCI non-severe pneumonia	N Amoxicillin or benzyl penicillin injection	% Amoxicillin or benzyl penicillin injection (95% CI)	N Cotrimoxazole	% Cotrimoxazole (95% CI)	N Other antibiotic	% Other antibiotic (95% CI)	N No antibiotic	% No antibiotic (95% CI)
	**Total**	**590**	**228**	**38.7 (33.0-44.7)**	**157**	**26.6 (21.7-32.1)**	**44**	**7.5 (5.0-11.1)**	**159**	**26.9 (21.3-33.4)**
**Patient or consultation characteristics**										
Fever complaint	No	164	63	38.6 (29.7-48.3)	43	26.1 (17.7-36.6)	17	10.2 (4.9-20.0)	39	28.4 (21.4-36.6)
	Yes	397	154	38.9 (32.1-46.2)	104	26.2 (20.6-32.6)	26	6.6 (3.8-11.2)	113	23.8 (14.6-36.2)
Diarrhea complaint	No	423	171	40.3 (34.5-46.5)	123	29.1 (23.6-35.2)	27	6.5 (3.8-10.9)	100	23.6 (18.5-29.6)
	Yes	166	58	34.7 (24.6-46.4)	34	20.4 (13.7-29.3)	17	10.0 (4.6-20.4)	58	35.0 (23.5-48.5)
Ear problem complaint	No	579	222	38.3 (32.4-44.6)	156	26.9 (22.0-32.4)	43	7.4 (4.8-11.0)	157	27.1 (21.2-33.9)
	Yes	11	6	57.8 (16.9-90.2)	1	10.0 (1.1-51.8)	1	13.1 (2.1-51.7)	2	19.1 (3.4-61.6)
Eye problem complaint	No	572	221	38.7 (33.0-44.6)	154	26.9 (22.0-32.5)	43	7.5 (4.9-11.2)	152	26.5 (21.3-32.4)
	Yes	18	7	38.4 (15.1-68.6)	3	14.7 (4.2-40.7)	1	6.7 (1.4-26.7)	7	40.2 (12.4-76.1)
Skin problem complaint	No	575	224	38.9 (33.2-45.0)	151	26.2 (21.2-31.0)	43	7.5 (4.9-11.0)	156	27.1 (21.3-33.0)
	Yes	14	4	27.8 (12.3-51.3)	6	42.7 (22.8-65.3)	1	7.4 (1.0-38.1)	3	22.1 (7.2-50.9)
Any danger sign complaint	No	307	121	39.5 (31.6-48.0)	85	27.6 (21.4-34.8)	21	7.0 (3.6-13.0)	78	25.6 (18.4-34.4)
	Yes	283	107	37.8 (30.3-45.0)	72	25.4 (19.2-32.0)	23	8.0 (4.2-14.0)	80	28.4 (21.7-36.0)
RDT done prior to consultation	No	358	139	38.7 (31.5-46.4)	86	23.9 (18.3-30.6)	27	7.5 (4.6-12.1)	105	29.3 (22.4-37.2)
	Yes	232	90	38.6 (30.8-47.1)	71	30.7 (23.7-38.7)	17	7.4 (3.5-14.0)	54	23.3 (17.1-31.0)
RDT result	Positive	78	26	33.5 (22.2-47.0)	15	18.9 (11.0-30.4)	3	3.5 (0.9-13.3)	34	44.1 (31.6-57.4)
	Negative	151	63	41.7 (32.2-51.0)	55	36.6 (26.7-40.0)	14	9.5 (4.2-19.0)	19	12.3 (7.4-19.0)
Temperature (Celsius)	37.5C or less	410	158	38.6 (31.9-45.6)	114	27.8 (21.9-34.6)	29	7.1 (4.6-11.0)	106	26.0 (20.0-32.9)
	37.6 – 38.9	144	50	34.8 (25.1-46.0)	36	25.1 (17.5-34.7)	14	9.8 (4.0-22.3)	44	30.2 (20.6-42.0)
	39.0 – 40.8	30	18	59.7 (41.9-70.0)	5	16.0 (6.9-30.0)	1	1.8 (0.2-10.0)	7	22.4 (10.9-40.0)
Child sex	Male	310	123	39.7 (32.3-47.6)	69	22.3 (16.7-29.2)	25	8.1 (4.9-13.3)	90	29.2 (21.3-38.7)
	Female	280	105	33.5 (30.3-45.0)	88	31.2 (24.8-38.5)	19	6.7 (3.4-12.9)	68	24.4 (18.3-31.0)
Child age (months)	2 – 11	166	76	45.9 (36.7-55.4)	29	17.5 (11.4-25.9)	14	8.7 (4.2-17.3)	45	27.2 (20.4-35.3)
	12 – 23	261	106	40.8 (31.9-50.4)	65	24.8 (18.7-32.2)	15	5.8 (3.2-10.0)	74	28.2 (18.6-40.4)
	24 – 35	88	30	33.8 (22.5-47.3)	35	40.3 (28.9-52.8)	3	4.0 (1.5-10.0)	19	22.0 (12.7-35.4)
	36 – 47	39	9	23.8 (13.0-39.5)	15	39.8 (21.9-60.9)	5	11.9 (5.0-25.8)	9	24.4 (13.2-40.7)
	48 – 59	37	7	18.3 (7.8-37.2)	12	33.1 (18.4-52.0)	6	17.4 (3.1-50.0)	12	31.2 (17.2-49.9)
Caregiver age (years)	11 – 19	58	19	33.2 (18.7-51.8)	14	23.6 (13.6-37.7)	3	4.7 (1.6-13.2)	22	38.5 (22.3-57.9)
	20 – 29	342	129	37.7 (30.7-45.2)	91	26.7 (20.5-34.0)	29	8.4 (5.3-12.9)	92	26.8 (21.3-33.1)
	30 – 39	151	64	42.3 (32.0-53.4)	40	26.5 (19.5-35.0)	9	6.1 (1.6-20.1)	37	24.5 (16.2-35.3)
	40 or more	20	9	46.2 (18.4-76.5)	7	35.9 (14.9-64.2)	2	8.6 (1.2-43.0)	2	9.3 (2.3-30.7)
Caregiver education	None	80	24	30.0 (19.9-42.6)	23	29.1 (18.6-42.4)	9	11.0 (2.8-34.6)	24	29.9 (17.5-46.1)

	Primary	350	136	38.7 (30.9-47.2)	101	28.7 (22.8-35.5)	16	4.5 (2.7-7.5)	98	28.0 (20.4-37.1)
	Secondary or more	130	51	39.0 (27.8-51.5)	28	21.2 (13.8-31.1)	17	13.3 (6.8-24.6)	34	26.5 (15.8-40.8)
Consultation start hour	7am – 10am	439	160	36.5 (30.4-43.1)	131	29.8 (24.7-35.5)	32	7.2 (4.5-11.3)	114	25.9 (20.0-32.9)
	11am – 2 pm	115	51	44.5 (31.0-58.8)	21	18.7 (11.4-29.3)	12	10.7 (4.2-20.0)	30	26.1 (16.0-39.7)
	3pm – 5 pm	36	17	46.3 (24.8-69.4)	4	11.6 (3.2-34.5)	0	0.0	15	42.0 (21.2-66.2)
Wait time reported (mins)	10 or less	165	60	36.2 (25.9-47.9)	27	20.1 (13.1-29.7)	32	12.5 (6.1-23.8)	89	31.0 (21.6-42.4)
	11 – 30	73	27	37.2 (27.3-48.4)	23	31.7 (21.7-43.7)	5	6.4 (2.2-17.3)	18	24.7 (16.6-35.0)
	31 – 60	81	32	39.3 (27.6-52.4)	26	32.2 (22.8-43.5)	3	3.4 (1.2-9.4)	20	25.1 (16.2-36.7)
	60 or more	226	89	39.7 (29.1-51.0)	61	27.2 (20.4-35.4)	15	6.5 (3.7-10.9)	59	26.1 (18.4-35.8)
Illness duration (days)	0 – 1	135	58	43.0 (31.6-55.2)	29	21.3 (13.9-31.3)	4	3.0 (1.2-7.3)	44	32.7 (22.3-45.1)
	2 – 4	363	130	35.8 (29.5-42.7)	104	28.8 (23.5-34.7)	35	9.7 (5.8-15.7)	92	25.4 (18.9-33.2)
	5 or more	92	40	43.5 (27.1-61.4)	24	25.5 (14.7-40.3)	5	5.3 (2.3-11.9)	23	24.5 (15.2-37.0)
First visit for current illness	First visit	555	213	38.3 (32.5-44.5)	150	27.0 (21.9-32.8)	42	7.5 (4.9-11.3)	150	27.0 (21.2-33.7)
	Follow-up visit	25	9	36.3 (21.3-54.7)	7	27.8 (14.7-46.2)	2	9.9 (3.3-26.1)	6	22.9 (11.0-41.7)
**Facility characteristics**										
Malaria endemicity ($PfPR_{2\text{-}10}$)	Under 0.20	448	164	36.7 (30.4-43.5)	121	27.1 (21.3-33.8)	41	9.1 (6.0-13.6)	120	26.9 (20.3-34.7)
	0.20 – 0.39	142	64	44.9 (32.8-57.7)	35	24.9 (16.8-35.3)	3	2.4 (0.7-7.3)	39	27.1 (17.8-38.9)
Transmission season	Off-peak	522	211	40.4 (34.1-47.0)	130	24.8 (19.7-30.8)	37	7.1 (4.4-11.2)	143	27.4 (21.2-34.6)
	Peak	68	18	25.7 (16.6-37.5)	27	39.9 (30.8-49.6)	7	10.4 (5.5-18.8)	16	23.5 (15.6-33.8)
Residence	Urban	170	83	49.2 (34.5-64.0)	9	5.6 (2.8-10.8)	17	10.2 (4.5-21.5)	59	34.8 (19.9-53.4)
	Rural	420	145	34.4 (29.3-40.0)	147	35.0 (30.1-40.3)	27	6.4 (4.3-9.4)	100	23.7 (19.6-28.4)
Facility type	Hospital	200	92	46.1 (32.8-59.9)	25	12.6 (6.2-24.1)	20	9.8 (4.5-19.9)	63	31.5 (18.4-48.4)
	Other facility type	390	136	34.9 (30.0-40.1)	131	33.7 (29.2-38.5)	25	6.3 (4.3-9.2)	96	24.6 (20.4-29.3)
Managing authority	Government	454	177	39.0 (32.0-46.5)	124	27.4 (21.4-34.4)	28	6.1 (3.3-10.8)	124	27.3 (20.3-35.5)
	CHAM or other	136	51	37.5 (29.9-45.8)	32	23.8 (17.4-31.6)	16	12.1 (7.4-19.0)	35	25.8 (19.4-33.4)
Region	North	68	25	35.9 (24.6-49.1)	20	28.9 (20.3-39.5)	3	5.0 (2.3-10.7)	21	30.1 (20.7-41.6)
	Central	317	132	41.8 (33.1-51.0)	75	23.7 (17.6-31.1)	13	4.3 (2.2-8.0)	95	30.0 (21.2-40.5)
	South	205	71	34.8 (26.3-44.4)	62	30.2 (20.7-41.8)	27	13.2 (8.1-20.9)	44	21.2 (14.8-29.4)
Any antibiotic available	No	1	0	0.0	1	100.0	0	0.0	0	0.0
	Yes	589	228	38.7 (33.0-44.7)	156	26.5 (21.6-32.0)	44	7.5 (5.0-11.1)	159	27.0 (21.3-33.5)
Amoxicillin available	No	10	0	0.0	5	54.2 (27.5-78.6)	2	21.0 (7.6-46.2)	2	24.8 (9.2-51.9)
	Yes	580	228	39.3 (33.5-45.4)	151	26.1 (21.2-31.7)	42	7.2 (4.7-10.9)	157	27.0 (21.3-33.6)
Timer available	No	32	14	42.7 (23.1-65.0)	7	21.2 (11.0-36.9)	2	7.1 (2.0-21.9)	9	29.0 (16.3-46.2)
	Yes	558	214	38.4 (32.5-44.7)	150	26.9 (21.8-32.7)	42	7.5 (4.9-11.3)	150	26.8 (20.9-33.6)
Total staff doctors	0	408	139	34.1 (29.4-39.1)	7	33.4 (29.0-38.2)	27	6.4 (4.4-9.3)	55	25.5 (21.3-30.2)
	1	31	7	22.9 (7.6-51.8)	11	36.7 (16.4-63.2)	6	18.6 (7.3-39.7)	7	21.8 (8.4-46.1)
	2 – 4	73	27	36.9 (17.7-61.3)	4	5.4 (1.7-15.4)	0	0.0	42	57.8 (31.2-80.5)
	5 or more	78	55	70.3 (46.8-86.5)	5	6.5 (0.8-37.1)	12	15.4 (6.0-34.4)	6	7.7 (1.7-28.9)
User fees (routine, general)	No	413	154	37.3 (29.9-45.2)	123	29.7 (23.6-36.6)	22	5.4 (2.9-9.8)	113	27.4 (19.9-36.3)
	Yes	177	74	42.0 (34.4-50.0)	34	19.1 (12.5-28.2)	22	12.3 (7.7-19.2)	46	25.9 (20.3-32.4)
Routine management meetings	No	59	27	45.1 (33.5-57.2)	13.0	21.9 (10.7-39.7)	10	16.4 (6.4-36.2)	10	16.5 (8.6-29.5)
	Yes	531	201	38.0 (31.8-44.6)	144.0	27.1 (21.9-33.0)	34	6.5 (4.2-9.8)	149	28.1 (22.0-35.1)
IMCI guidelines available	No	202	75	36.9 (28.5-46.2)	47	23.2 (15.2-33.8)	27	13.5 (8.3-21.3)	52	25.8 (18.5-34.8)

	Yes	388	154	39.6 (32.2-47.5)	110	28.3 (22.6-34.8)	17	4.3 (2.6-7.1)	107	27.5 (20.1-36.4)
**Provider characteristics**										
Job qualification	Doctor	67	39	58.7 (44.1-71.9)	10	15.4 (8.0-27.7)	7	11.1 (2.8-34.9)	10	14.8 (7.6-26.8)
	Medical assistant	436	161	36.9 (29.9-44.5)	116	26.5 (20.8-33.1)	31	7.0 (4.4-11.2)	127	29.1 (22.0-37.4)
	Nurse or other	87	28	32.2 (23.3-42.6)	31	35.5 (26.8-45.2)	6	6.8 (3.4-13.2)	22	25.5 (18.0-34.9)
Year qualification received	Before 2000	136	43	31.9 (22.5-43.1)	34	25.5 (17.4-35.7)	7	5.0 (2.6-9.3)	51	37.4 (22.7-54.9)
	2000 – 2009	183	62	33.8 (26.1-42.4)	59	32.5 (25.7-40.1)	13	6.9 (3.7-12.6)	48	26.2 (19.5-34.2)
	2010 to present	238	110	46.1 (36.1-56.5)	59	24.6 (17.5-33.5)	18	7.4 (3.6-14.8)	51	21.5 (14.5-30.6)
Provider sex	Male	395	150	38.1 (31.0-45.8)	103	26.2 (20.9-32.2)	29	7.4 (4.5-12.0)	109	27.7 (20.5-36.3)
	Female	162	64	39.8 (29.8-50.7)	49	30.4 (21.4-41.3)	8	4.9 (2.5-9.4)	40	24.9 (17.1-34.6)
Supervisor or in-charge	No	264	117	44.4 (33.9-55.5)	52	19.7 (13.2-28.4)	22	8.3 (4.4-15.1)	72	27.3 (16.9-40.8)
	Yes	292	97	33.4 (27.9-39.3)	101	34.4 (29.3-39.8)	15	5.2 (3.1-8.5)	78	26.6 (21.7-32.1)
IMCI in-service training	None	270	118	43.6 (34.5-53.2)	66	24.5 (18.5-31.6)	20	7.6 (4.0-13.7)	65	24.0 (17.1-32.5)
	Ever received	287	97	33.9 (27.2-41.4)	87	30.2 (23.6-37.7)	17	5.9 (3.4-9.8)	85	29.7 (21.2-39.8)
Recent supervision	None	100	43	42.9 (28.0-59.1)	20	20.2 (12.2-31.6)	4	4.3 (1.6-11.0)	32	31.8 (18.8-48.5)
	Past 3 months	379	152	40.2 (33.6-47.2)	111	29.1 (23.3-35.8)	26	7.0 (4.1-11.7)	88	23.3 (16.5-31.9)
	Over 3 months ago	77	19	25.3 (15.6-38.3)	22	28.3 (19.1-39.8)	6	8.2 (4.0-16.2)	29	38.2 (26.8-51.0)
